# Large complex odontomas associated with impacted mandibular second and third molars: a case report

**DOI:** 10.1186/s13256-026-06166-w

**Published:** 2026-06-06

**Authors:** Elham Babadi, Hassan Mir Mohammad Sadeghi, Mohammad Mehdi Rahmani, Fatemeh Mashhadiabbas

**Affiliations:** Department of Oral and Maxillofacial Surgery, Dental School, Shahid Beheshti University of Medical Science, Tehran, Iran

**Keywords:** Odontoma, Odontogenic tumor, Mandible, Facial asymmetry, Hamartoma

## Abstract

**Background:**

Odontomas are the most common odontogenic tumors and are composed of dental tissues such as enamel, dentin and cementum. They are classified into compound and complex types. Compound odontomas usually appear as tooth-like structures in the anterior maxilla, whereas complex odontomas present as irregular masses of dental tissues, most frequently in the posterior mandible. Although odontomas are generally slow-growing and asymptomatic, large lesions can cause jaw expansion, facial asymmetry, and delayed tooth eruption. Various surgical approaches have been described, ranging from intraoral to extraoral incisions, with or without fixation.

**Case presentation:**

In the present study, we present a 21-year-old Iranian woman with a large mandibular complex odontoma extending from the first molar to the ramus. The lesion was removed via an intraoral approach without pathological fracture or the need for fixation. At the 4-month follow-up, the patient experienced uneventful healing and no signs of recurrence.

**Conclusion:**

This case highlights that even large mandibular odontomas can be managed intraorally without the need for fixation devices, with favorable short-term outcomes.

## Introduction

Odontoma is the most prevalent odontogenic lesion of the maxilla and mandible. Although historically classified as a tumor, it is now considered a hamartoma that develops from odontogenic epithelium and ectomesenchyme components and has the capacity to form enamel, dentin and cementum [1]. Most cases are asymptomatic and are often discovered incidentally during routine radiographic examinations [2]. No specific etiology for odontoma has been identified; however, several factors, such as trauma to primary dentition, remnants of Malassez, inflammatory processes, odontoblastic hyperactivity, and hereditary anomalies, have been suggested [3].

These lesions occur equally in men and women, most commonly in the second and third decades of life. They are divided into two main types: compound odontomas, which are composed of multiple tooth-like structures and are usually located in the anterior maxilla, and complex odontomas, which present as irregular disorganized masses of dental tissue that are often found in the posterior mandible [4, 5]. Complex odontomas can erupt into the oral cavity, but their eruption differs from that of normal teeth, as they lack a periodontal ligament. Bone sequestration or remodeling may also occur as the lesion develops. They are usually asymptomatic when small, but as they grow, they can cause jaw expansion and facial asymmetry [2]. Radiographically, complex odontomas appear as radiopaque or calcified dental masses with irregular shapes that are dissimilar to those of normal teeth [6].

In the present study, we present a rare case of a large complex odontoma of the mandible that limited the eruption of the second and third molars and caused cortical expansion with intraoral exposure.

## Case presentation

### Patient description

**Fig. 1:**
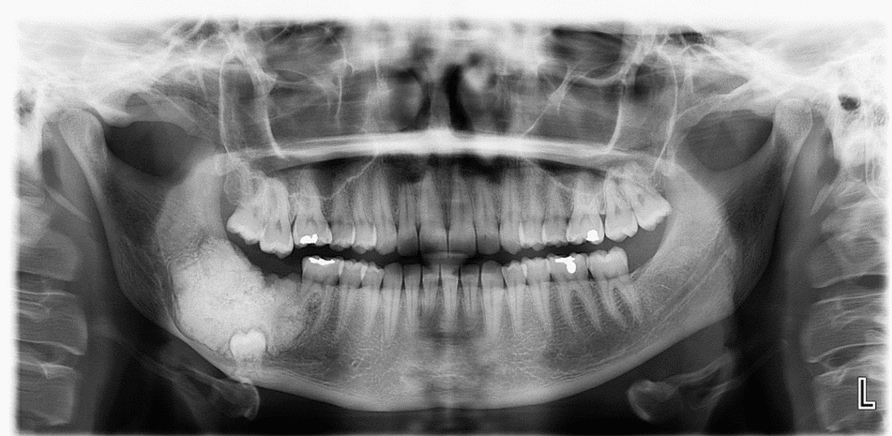
Preoperative panoramic radiography

**Fig. 2:**
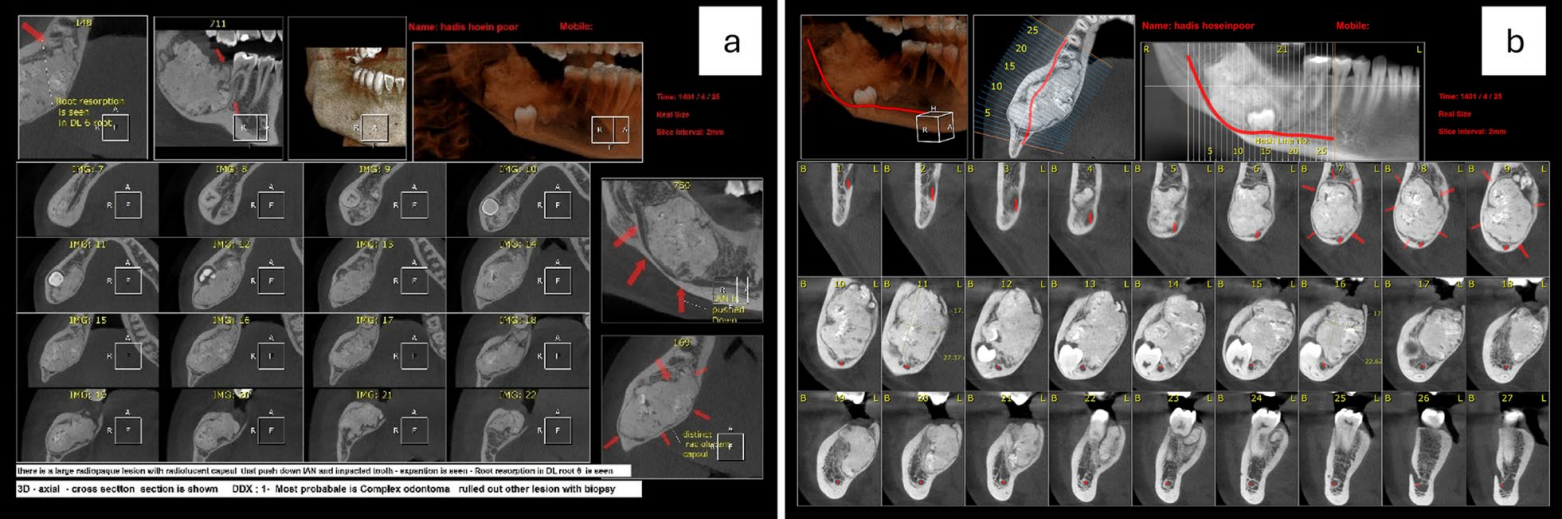
Preoperative cone-beam computed tomography. **A** Axial, **B** sagittal

**Fig. 3:**
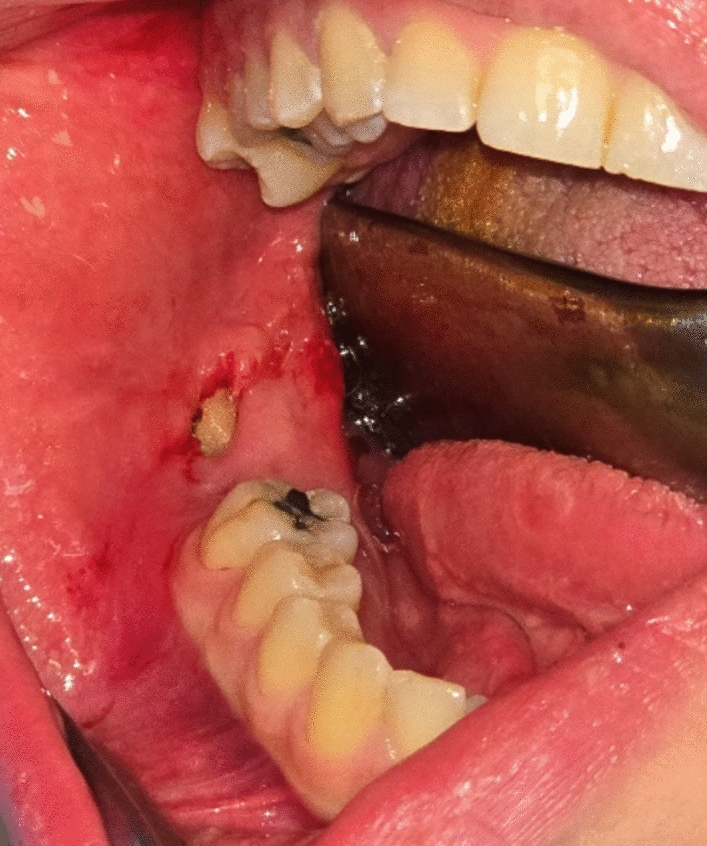
Preoperative intraoral photography

A 21-year-old Iranian woman was referred to Imam Hossein Hospital in March 2025 with the chief complaints of pain and swelling in the right mandibular angle. The patient reported that approximately two years earlier, she accidentally noticed the pathologic lesion and impacted teeth on a panoramic radiograph but did not seek treatment at that time. The patient had no history of any disease or medication use. She also did not report any similar conditions in her family. Motor and sensory nerve functions were within normal limits. Panoramic radiography and cone-beam computed tomography (CBCT) were performed. The panoramic radiograph revealed a radiopaque lesion with a thin radiolucent rim extending from the right first mandibular molar to the ascending ramus (Fig. 1). On CBCT, the lesion involved the inferior alveolar canal and displaced it inferiorly. Additionally, it displaced the third molar toward the inferior border of the mandible and obstructed eruption of the second molar, leading to severe resorption of the second molar and the distal root of the first molar. The lesion caused bone expansion and thinning of the buccal and lingual cortical plates (Fig. 2). An incisional biopsy was performed, and histopathological examination confirmed the diagnosis of a complex odontoma. During oral examination, a part of the occlusal aspect of the lesion was visible in the retromolar pad area (Fig. 3).

### Treatment plan

**Fig. 4:**
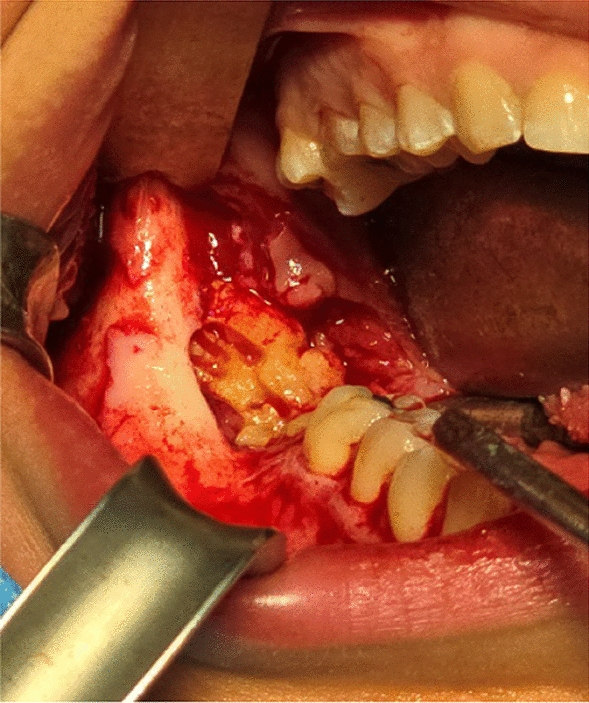
Intraoperative photography

**Fig. 5:**
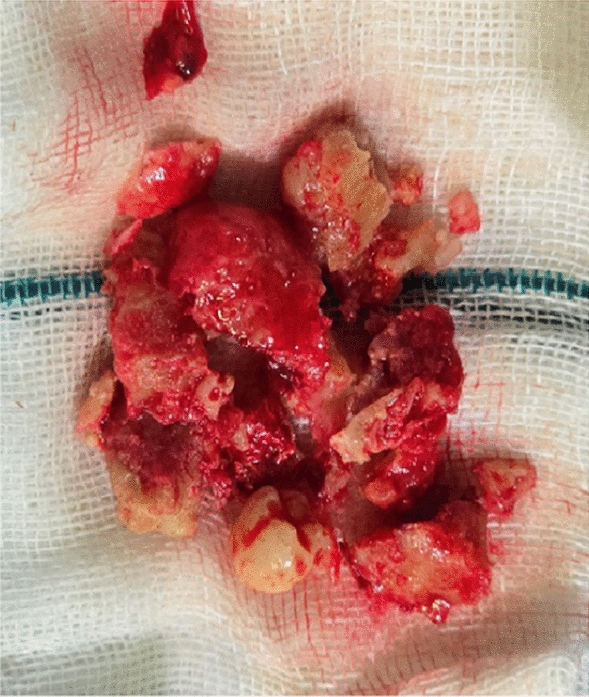
Biopsy specimen

**Fig. 6:**
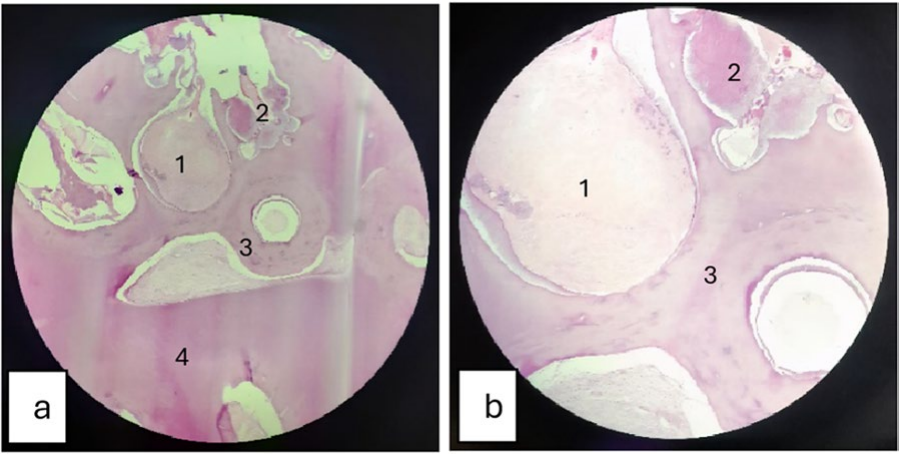
Microscopic slides. **A-1** Pulp, **A-2** enamel matrix, **A-3** cementum, **A-4** dentin, **B-1** pulp, **B-2** enamel matrix, **B-3** dentin

Surgical treatment, consisting of excisional biopsy of the lesion, along with extraction of the first, second, and third molars, was planned. A sulcular incision was made, extending from the mesial aspect of the right mandibular canine to the anterior border of the ramus, with posterior and anterior releasing incisions. After flap reflection, the lesion was clearly visible in the distal to the second molar (Fig. 4). The first molar was extracted via an atraumatic technique. The lesion was then segmented and gradually removed to avoid damaging the buccal and lingual cortical plates as well as the inferior border of the mandible. The second and third molars were removed along with the lesion (Fig. 5). An absorbable gelatin sponge was placed in the cavity, and the flap was repositioned and closed via 3-0 resorbable sutures, achieving primary closure. Cefazolin (1 g/intravenous/*quater in die* (QID)) was administered for 3 days, after discharge, Cephalexin (500 mg/per oral/QID) was prescribed for one week. The excised lesion and extracted teeth were sent to the pathology department of Shahid Beheshti Dental School, and the final histopathological diagnosis was complex odontoma. Microscopic examination revealed dental structures containing dentin-like tissue with irregular tubular architecture. In areas of the odontogenic epithelium, cells resembling ameloblasts that produce an enamel matrix were observed, along with cementum globules and pulp-like spaces (Fig. 6). During the 4-month follow-up, the patient experienced uneventful healing, and no signs of recurrence were seen at panoramic radiography.

## Discussion

Odontomas, the most prevalent odontogenic tumors, are composed of different dental tissues. In compound odontomas, these tissues form tooth-like structures, whereas in complex odontomas, they exhibit a disorganized pattern. These lesions occur equally in males and females, with an age range of 20–30 years. The most common site for compound types is the anterior maxilla, whereas complex types are more frequently found in the posterior mandible [5]. Our patient was a 21-year-old woman with a large complex odontoma that extended from the first right molar to the ramus. The lesion caused expansion of the jawbone and thinning of the buccal and lingual cortical plates.

Odontomas usually have limited growth; however, in rare cases of complex odontomas, the lesion exceeds 3 cm. Large odontomas can restrict tooth eruption, displace the inferior alveolar canal or teeth, limit mouth opening, and become exposed to the oral cavity, causing root resorption, pain, swelling and facial asymmetry [7–11]. In our case, the odontoma prevented eruption of the second and third molars and pushed the inferior alveolar nerve inferiorly. Resorption by the roots of the first molar was also observed. Moreover, the eruption of the odontoma led to exposure of the oral cavity at the retromolar pad area.

Several differential diagnoses must be considered, including osteomas, ameloblastic fibro-odontomas, odontoameloblastomas, cementoblastomas, and fibro-osseous lesions such as cemento-osseous dysplasia. Osteomas can be radiographically distinguished from odontomas by the lack of radiolucent rims around the former. Cemento-osseous dysplasia typically develops around root apices in patients over 30 years of age [8]. Ameloblastic fibro-odontomas reveal radiolucencies that contain radiopaque components and therefore can be historically and radiographically indistinguishable from odontomas [8, 9]. Odontoameloblastomas present multilocular radiolucency with some radiopaque components and involve patients under 20 years of age. Park et al. reported that the main difference between mixed odontogenic tumors such as ameloblastic fibro-odontoma or odontoameloblastoma and odontoma is their growth potential [8]. Cementoblastomas occur in the periapical area of the roots and involve the periodontal ligament space, which may mimic the radiolucent rim observed around odontomas [12]. Comprehensive clinical and radiographical examinations as well as incisional biopsy are essential for accurate diagnosis and treatment planning. In our case, the diagnosis was confirmed by the pathological report of the incisional biopsy.

Different surgical approaches have been reported for the removal of lesions, including intraoral or extraoral incisions, with or without plate fixation. Most case reports have used intraoral access and enucleation of the lesion, along with extraction of the involved teeth [7, 11, 13–15]. Park et al. [8] accessed the lesion via an intraoral approach and reconstructed the remaining defect with iliac bone grafts. They fixed the graft with mini plates and applied maxillomandibular fixation (MMF) via skeletal anchorage screws and rubber for one week. Neto et al. [10] used intraoral access and a prebent reconstruction plate to prevent possible pathological fracture. Christopher et al. [16] interestingly used split sagittal osteotomy (SSO) to access the lesion. They mentioned that this technique is useful for deep or lingually placed lesions while protecting the buccal and lingual cortical plates. However, one disadvantage of this approach is the need for fixation plates and screws and MMF for two weeks. Some oral and maxillofacial surgeons prefer extraoral approaches such as the Risdon incision to access the lesion [9, 12]. Despite the disadvantages of these methods, such as scarring and potential injury to the marginal mandibular nerve, they provide appropriate visibility and access to the lesion. As esthetics were important in our case and since the lesion had erupted into the oral cavity, we opted for an intraoral approach. The lesion caused expansion and thinning of the buccal and lingual cortical plates; therefore, adapting fixation plates and using screws was difficult and unnecessary in our case.

Histopathological confirmation is critical, particularly when an incisional biopsy has not been performed before surgery. Additionally, long-term follow-up is recommended to monitor for recurrence and detect complications such as pathological fracture.

## Conclusion

Odontomas are benign odontogenic tumors that are often incidentally detected during routine radiographic examinations. Early diagnosis and treatment of these lesions is easier and can help prevent complications, including the involvement of surrounding structures and facial asymmetries. In selected cases, large mandibular odontomas can be successfully treated intraorally without fixation. Patients were advised to avoid facial trauma, maintain a soft diet, and attend regular follow-up visits.

## Data Availability

Not applicable.
